# Autophagy-lysosome pathway in insulin & glucagon homeostasis

**DOI:** 10.3389/fendo.2025.1541794

**Published:** 2025-02-10

**Authors:** Yi Wu, Hui Wang, Huoyan Xu

**Affiliations:** ^1^ School of Pharmacy, Shanghai University of Medicine and Health Sciences, Shanghai, China; ^2^ Shanghai University of Medicine & Health Sciences Affiliated Zhoupu Hospital, Shanghai, China; ^3^ Shanghai Key Laboratory of Molecular Imaging, School of Pharmacy, Shanghai University of Medicine and Health Sciences, Shanghai, China

**Keywords:** lysosome, autophagy, insulin homeostasis, glucagon homeostasis, diabetes

## Abstract

Lysosome, a highly dynamic organelle, is an important nutrient sensing center. They utilize different ion channels and transporters to complete the mission in degradation, trafficking, nutrient sensing and integration of various metabolic pathways to maintain cellular homeostasis. Glucose homeostasis relies on tightly regulated insulin secretion by pancreatic β cells, and their dysfunction is a hallmark of type 2 diabetes. Glucagon also plays an important role in hyperglycemia in diabetic patients. Currently, lysosome has been recognized as a nutrient hub to regulate the homeostasis of insulin and other hormones. In this review, we will discuss recent advances in understanding lysosome-mediated autophagy and lysosomal proteins involved in maintaining insulin and glucagon homeostasis, as well as their contributions to the etiology of diabetes.

## 1 Introduction

Lysosomes, first were discovered by Christian De Duve (1), are highly acidic compartments surrounded by a single lipid bilayer. The high acidity of the lysosomal lumen is maintained by the vacuolar H+-ATPase (v-ATPase). There are more than 60 different lysosomal hydrolases including lipases, proteases, and glycosidases that degrade various substrates such as extracellular or cell surface cargos from endocytosis and intracellular components from autophagy (2, 3). Thus, for a long time, lysosomes were regarded primarily as the major degradation center in the cell. However, evidences accumulated to show that lysosome could be a hub of nutrient sensors to receive inputs from various nutrient stimuli or growth factor/hormone signaling and send outputs to control cellular metabolism in healthy and diseased states (3, 4). As reported, to adapt to dynamic cellular environments, lysosomes are equipped with nutrient-sensing machinery, including the mechanistic/mammalian target of rapamycin (mTOR) complex, a master regulator of cell growth, and its associated proteins (5–7).

Lysosomes play a central role in degrading substrates delivered via secretory, endocytic, phagocytic, and autophagic pathways. The major one is autophagy. Autophagy not only promotes cell survival after starvation but also is important under basal or nutrient excess conditions. Cytosolic components are captured by autophagosome and then fused with lysosomes to initiate the degradation of its contents. Autophagy can provide essential components for energy production and biosynthesis under nutrient depletion condition and also play a vital role in recycling damaged organelles, unnecessary proteins, and foreign substances to maintain cell normal function (8).

Pancreatic islets mainly contain glucagon-producing α cells and insulin producing β cells (9). Insulin is a key hormone to control blood glucose in response to glucose while glucagon counteracts the actions of insulin to maintain glucose level. Upon chronic nutrient overload, such as those associated with obesity and overnutrition, insulin release will be increased through several adaptive cellular mechanisms. However, when these mechanisms are overwhelmed, β cells eventually fail, leading to type 2 diabetes mellitus (T2DM), which is characterized by chronic hyperglycemia, insulin resistance in peripheral metabolic tissues and inadequate insulin secretion from β cells (3, 10, 11).

Various lysosomal enzymes are implicated in insulin secretion (12–16), and the degradation of insulin requires normal lysosome function. Autophagy plays vital roles in supporting basic function of β cells. In the pathology of T2DM, autophagy activity is initially elevated and protective in β cells during the prediabetic stage but decreases as the disease progresses, contributing to β cell failure (17, 18). Autophagy is mTOR dependent, while other channels, such as TPC2 and P2X4, also play significant roles. Beyond insulin regulation, autophagy is also involved in maintaining glucagon homeostasis (19–21).

As lysosomes and autophagy interact to maintain cellular nutrient balance and regulate insulin and glucagon homeostasis, understanding their functional components and regulatory networks could provide valuable insights into diabetes pathology and pave the way for developing therapeutic strategies to treat the disease.

## 2 Lysosome

Lysosomes utilize different channels and signaling pathways to regulate lysosomal ion homeostasis, membrane potential, catabolite export, membrane trafficking and sense nutrient changes. They are distributed throughout the body, and their dysfunction is linked to numerous diseases, including lysosomal storage disorders (LSDs), abnormal embryonic development, and cancer (3, 4, 22).

### 2.1 Important lysosomal proteins and channels

#### 2.1.1 mTOR

mTOR is a key serine/threonine kinase in sensing nutrients changes and integrating environmental inputs into downstream signaling pathways to control cellular metabolism, growth, and survival upon metabolic and nutrient changes. It is named so because mTOR can be inhibited by rapamycin, a compound produced by a Gram-positive soil bacterium found on island called ‘‘Rapa Nui’’ (23). mTOR is found in two protein complexes: mTOR Complex 1and mTOR Complex 2 (mTORC1 and 2). mTORC1 is composed of mTOR, Raptor, PRAS40, Deptor, and mLST8/GbL while mTORC2 is composed of mTOR, mLST8/GbL, Deptor, Rictor, mSin1, and Protor. mTORC1 is well studied, however, less is known about mTORC2 (24, 25).

**Figure 1:**
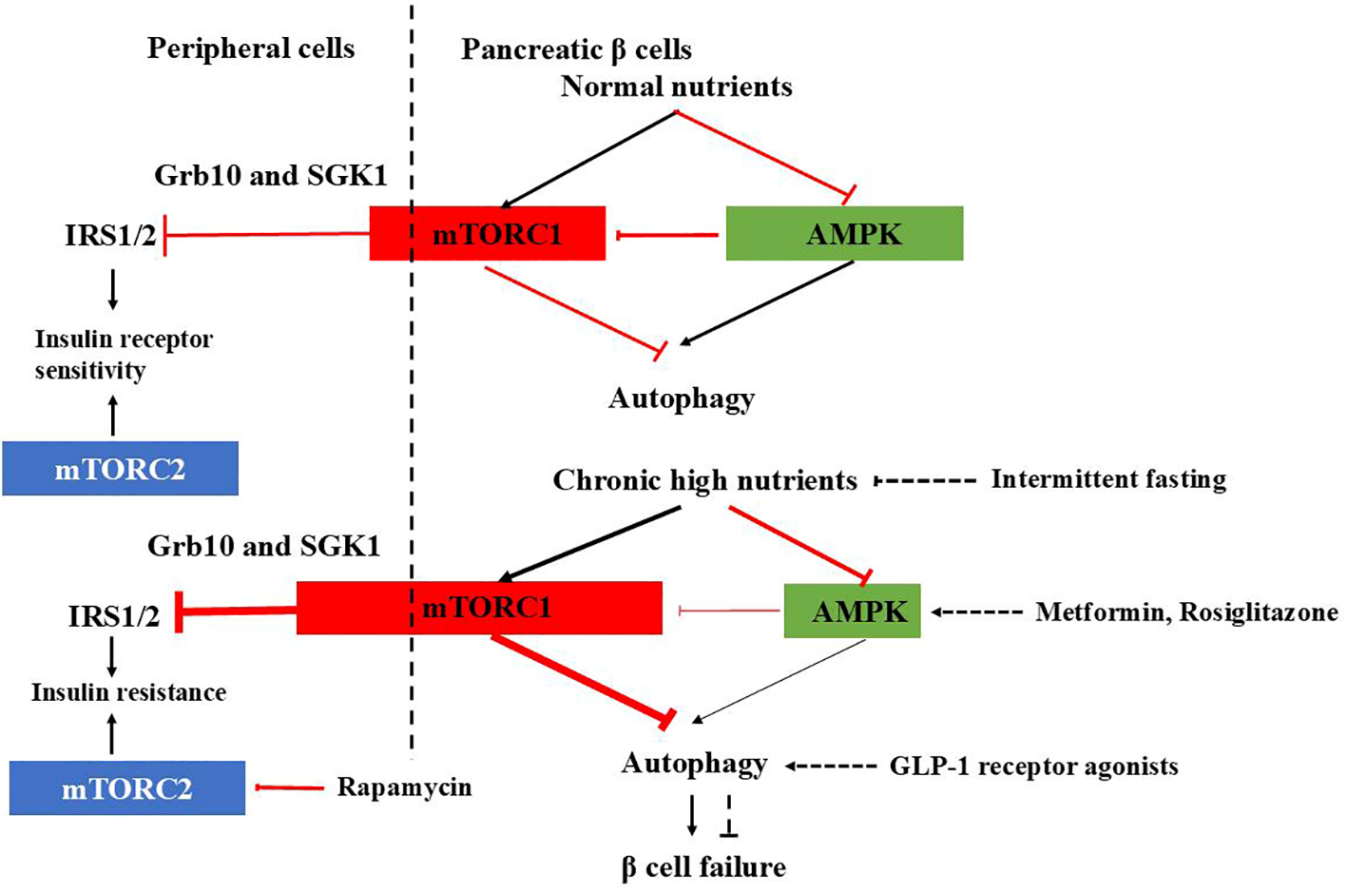
mTORC1-AMPK signaling in pancreatic β cells and peripheral cells under physiological and T2DM conditions and therapeutic agents affecting autophagy. In normal β cells, autophagy is tightly regulated by mTORC1-AMPK signaling, ensuring proper insulin release. Under healthy conditions, mTORC1 inhibits IRS1/2 through Grb10 and SGK1 to adjust insulin sensitivity and prevent excessive insulin actions in the periphery. However, in T2DM, the check and balance are disturbed. In β cells, chronic high nutrition activates mTORC1 abnormally and autophagy is suppressed, causing β cells failure. In the periphery, overactivated mTORC1 inhibits IRS1/2, leading to insulin resistance. mTORC2 promotes glucose uptake, glycolysis, and cell survival, inhibition of which leads to insulin resistance. Therapeutic agents affecting autophagy to improve diabetes are also shown.

When nutrients such as amino acids, glucose, cholesterol, and nucleotides are abundant, or cellular energy levels are high, mTORC1 becomes activated. The lysosomal v-ATPase senses the high concentration of amino acids within the lysosome. Then, v-ATPase interacts with Regulator-Rag complex to trigger guanine nucleotide exchange factor (GEF) activity of Regulator towards RagA/B. Rag GTPases is a heterodimer consisting of Rag A/B bound to Rag C/D. These specific conformational changes allow the binding of rags to the raptor subunit of mTORC1 recruiting mTORC1 to the lysosomal membrane. Finally, Rheb protein activates mTOR at the lysosomal surface (7, 26, 27). Conversely, when amino acid levels inside the lysosome are low, the change in conformation of the Regulator and Rag complexes promotes the recruitment and phosphorylation of the tuberous sclerosis complexes (TSC) which is mTORC1 negative upstream regulator (28–30). Then the Rheb-GAP activity of TSC is increased which converts active GTP-Rheb to inactive GDP- Rheb impeding the stimulatory effects of Rheb on mTORC1 (29). Ultimately, mTORC1 activation enhances protein translation by phosphorylating p70S6 Kinase 1 (S6K1) and eIF-4E-binding protein 1 (4E-BP1) (25, 31), which promotes protein synthesis. This activation drives cell growth and proliferation and plays a significant role in tumor metabolism as shown in **Figure 1**.

Glucose depletion can inhibit mTORC1 through AMPK-dependent or -independent mechanisms (32) (**Figure 1**). AMPK inhibits mTORC1 in response to glucose starvation either through the phosphorylation and activation of the TSC2 (33) or through the phosphorylation and inhibition of Raptor (34). However, mTORC1can also sense glucose level through AMPK-independent way (35–39). The anti-diabetic drug metformin was found to inhibit mTORC1 in a Rag GTPase-dependent manner but independent of TSC1/2 and of AMPK (34). A glycolytic intermediate dihydroxyacetone phosphate (DHAP) is sensed via a mechanism that is dependent upon GATOR–Rag signaling but independent of AMPK (35). GLUT1, which facilitates the diffusion of glucose across cell membrane, has been found to enhance mTOR activity independently of TSC2 and AMPK (39). GLUT1 inhibition by small-molecule inhibitor compounds selectively and potently dampen the mTORC1 activity (40). Moreover, glucose influences mTORC1’s assembly, lysosomal localization, and overall activity, either directly or indirectly (41, 42).

In addition to mTORC1, mTORC2 is also involved in carbohydrate metabolism. Extensive evidence demonstrates that mTORC2 promotes glucose uptake, glycolysis, and cell survival through the activation of members of the AGC kinase family, such as Akt, serum/glucocorticoid-regulated kinase (SGK), and protein kinase C (PKC) (43–49). So, dysfunction of mTORC2 signaling has been implicated in the development of insulin resistance and diabetes. For instance, chronic treatment with rapamycin induces glucose intolerance, primarily by causing hepatic insulin resistance. Moreover, genetic disruption of hepatic mTORC2 alone is sufficient to replicate this effect. Similarly, disruption of mTORC2 in various tissues, including adipose tissue, brain, pancreatic islets, and skeletal muscle, leads to glucose intolerance and impaired insulin sensitivity (48) (**Figure 1**).

#### 2.1.2 TPC channels

Two-pore channels(TPCs), including TPC1 and TPC2, are ubiquitously expressed in the endolysosomal system of mammalian cells (50). While TPC1 is expressed in both early endosomes (EEs) and lysosomes, TPC2 is predominantly present on the lysosomal membranes (51). These channels are highly Na^+^ selective channels with minimal Ca^2+^ permeability (52). TPCs can be modulated by several factors, including nicotinic acid adenine dinucleotide phosphate (NAADP), phosphatidylinositol 3,5-bisphosphate (PI(3,5)P2), and extracellular nutrients (52, 53). Interestingly, in rats, the expression levels of TPC2 are negatively correlated with fasting glucose levels, suggesting a potential role for TPC2 in maintaining normal glucose homeostasis (54).

#### 2.1.3 P2X4 channels

The P2X4 channel is a trimeric, two-transmembrane (2TM) ion channel that belongs to the ionotropic P2X-family of ATP receptors. Upon activation by ATP, it becomes permeable to both Na^+^ and Ca^2+^. The activation of lysosomal P2X4 channels by luminal ATP is pH-dependent: when the luminal side is acidic, P2X4 activity is inhibited. P2X4 channels play a specific role in promoting endolysosomal membrane fusion by activating calmodulin through calcium release from P2X4 channel (55, 56). Additionally, the involvement of P2X4 channels in maintaining insulin homeostasis has also been reported (57).

#### 2.1.4 TRPML channels

Members of the Transient Receptor Potential Mucolipin (TRPML) channel subfamily—TRPML1, TRPML2, and TRPML3—are primarily localized in endosomes and lysosomes. These TRPML channels are cation channels permeable to Ca^2+^ Na^+^, K^+^, Fe^2+^, and Zn^2+^. While TRPML1 is ubiquitously expressed across various tissues, TRPML2 and TRPML3 are more selectively expressed in specific organs. TRPML channels play crucial roles in membrane trafficking, autophagy, lysosomal biogenesis, and lysosomal exocytosis (58). Notably, lysosomal calcium release via TRPML1 is essential for the activation of mTORC1. Depletion of TRPML1 inhibits mTORC1 activity, whereas its activation promotes mTORC1 signaling (59). When nutrients are abundant, mTORC1 phosphorylates and inhibits TRPML1. However, during starvation, mTORC1-mediated inhibition is relieved, leading to the activation of TRPML1. In turn, activated TRPML1 enhances mTORC1 activity, a process that requires calmodulin (CaM). Both TRPML1 and CaM are essential for the reactivation of mTORC1 during prolonged starvation. Therefore, TRPML1 serves as a negative feedback regulator of mTORC1, preventing excessive loss of mTORC1 function, which is critical for maintaining cellular homeostasis during periods of nutrient deprivation (60). Loss-of-function mutations in human TRPML1 result in type IV mucolipidosis (ML-IV), an autosomal recessive lysosomal storage disorder (61).

#### 2.1.5 TRPM channels

TRPM (Transient Receptor Potential Melastatin) channels are expressed in the plasma membrane and various cell organelles. The TRPM family consists of TRPM1 through TRPM8. TRPM channels are highly permeable to divalent cations such as calcium, magnesium, and zinc (TRPM1, TRPM3, TRPM6, and TRPM7), as well as nonselective cations (TRPM2 and TRPM8) (62). Among them, TRPM2 has been shown to be expressed in lysosomes of pancreatic β cells and dendritic cells (63, 64). TRPM2 is a Ca^2+^-permeable channel that contains a unique adenosine diphosphoribose (ADPR) hydrolase domain in its C-terminus. It is thermosensitive and is synergistically activated and regulated by ADPR (including ADPR analogs), hydrogen peroxide (H_2_O_2_), and intracellular calcium (63, 65, 66). TRPM2 may regulate autophagy in pericytes and cancer cells, potentially influencing the early stages of autophagy (67, 68). However, recent reports suggest that TRPM2 can also facilitate lysosomal acidification, leading to excessive autolysosome degradation and subsequent cell death in primary cultured mouse aortic smooth muscle cells (69).

#### 2.1.6 Other channels and proteins

Other channels and proteins in the lysosome including TMEM175, a K^+^ and Cs^+^ selective channels on lysosomes to maintain lysosomal membrane potential (70) and solute carrier family 38 member A7 (SLC38A7), acting as amino acid transporter (71).

### 2.2 Function of lysosome

Lysosomes receive cargo primarily from the endocytic and autophagic pathways. In endocytosis, extracellular substrates are initially delivered to early endosomes, which mature into late endosomes (LEs). LEs subsequently fuse with lysosomes to form endolysosomes (ELs). In the autophagic pathway, damaged intracellular material is enclosed within autophagosomes (APs), which also fuse with lysosomes to form autolysosomes (ALs) (72). Within ELs and ALs, lysosomal hydrolases degrade the endocytic and autophagic substrates (73, 74). After degradation, the resulting digested products are transported out of the lysosome, either through specific catabolite exporters or via vesicular membrane trafficking mechanisms. Apart from degrading the foreign and intracellular material by endocytosis and autophagy, lysosome also kill and degrade pathogens through phagocytosis that are engulfed by it and also involves in antigen processing in antigen presenting cells like dendrite cells and macrophages (75). However, in this review we focus on autophagy as it plays an important role in insulin and glucagon homeostasis.

Currently, three major types of autophagy have been identified: macroautophagy, microautophagy, and chaperone-mediated autophagy (CMA). Additionally, several less common forms have been characterized, including crinophagy, in which secretory vesicles degrade their contents by fusing directly with lysosomes. This process is particularly relevant in exocrine, endocrine, and neuroendocrine cells. Other notable types include starvation-induced nascent granule degradation (SINGD), Golgi membrane-associated degradation (GOMED), and vesicophagy. Given the role of β cells as hormone-producing cells, these forms of autophagy may also contribute to their function and regulation (76–79).

Unlike conventional autophagy, microautophagy involves the direct internalization of cytoplasmic component into lysosomes by membrane invagination. It plays important roles in organelle remodeling, membrane homeostasis, and cell survival when cells are under stress (80). However, little is known about this process, especially its physiological function and regulation in mammalian cells. Chaperone-mediated autophagy differs from microautophagy and microautophagy in that it does not require formation of vesicles. Instead, the translocation of substrates for CMA requires selectively binding of molecular chaperone proteins. Under starvation conditions, CMA will be upregulat2ed to overcome nutrient stress (81, 82).

**Figure 2:**
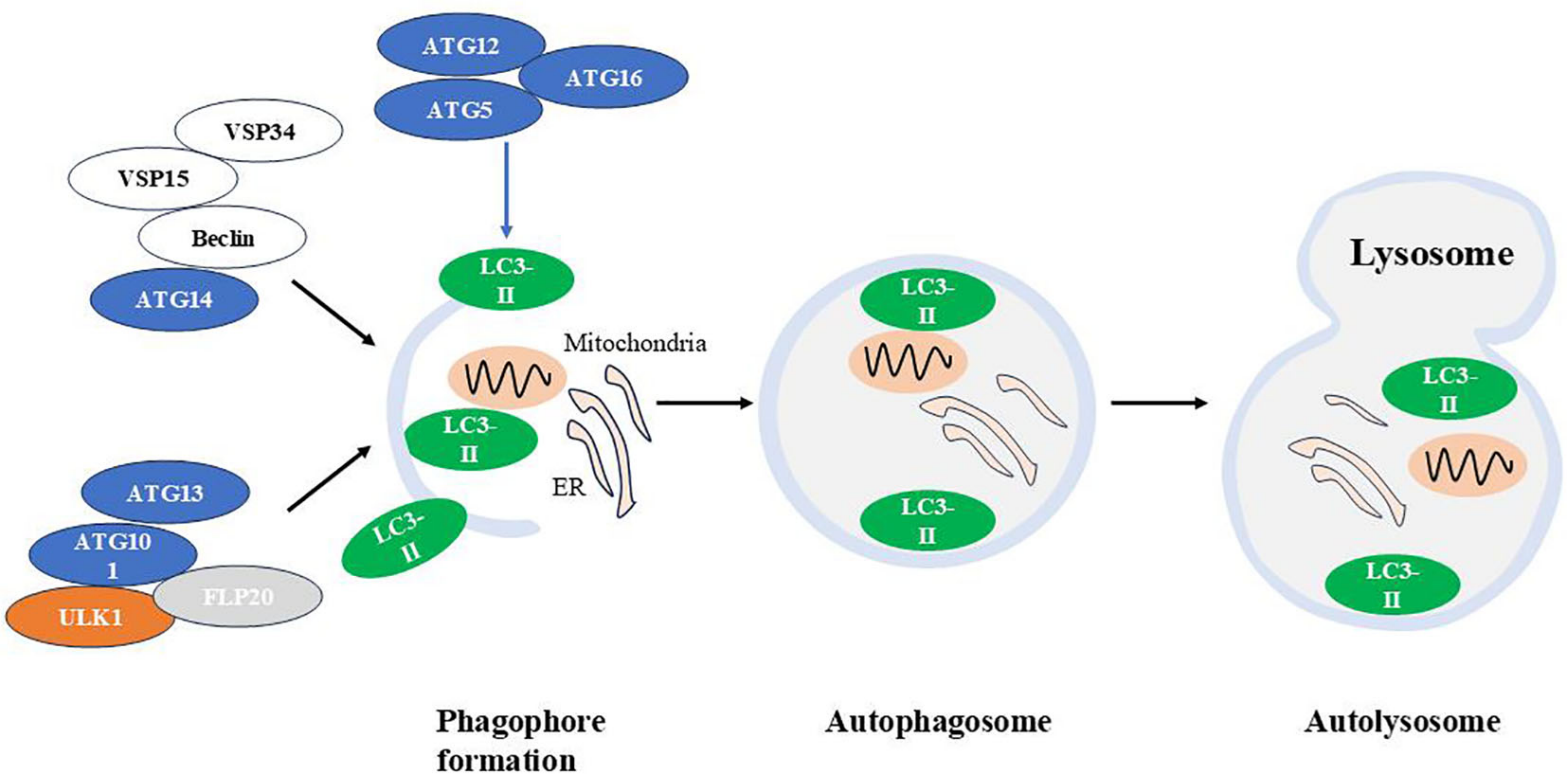
The autophagic machinery. Autophagy is a cellular process that involves the formation of autophagosomes, their fusion with lysosomes, and the subsequent degradation of autophagolysosomal contents. *Atg*, autophagy-related gene; LC3, Light chain 3.

Macroautophagy degrades substrates such as bulk cytoplasm, organelles, aggregate-prone proteins, and infectious agents. This autophagic process has four stages: initiation, nucleation, elongation, and fusion/degradation (83). It begins with the formation of a U-shaped double-layer lipid membrane, or phagophore, which originates from the omegasome at the endoplasmic reticulum (ER) and/or mitochondria-associated ER membranes (MAMs). Once released, the phagophore engulfs cellular components, expanding to form a closed structure known as the autophagosome. The mature autophagosome then fuses with the lysosome to create an autolysosome, initiating protein degradation (10, 84) see **Figure 2**.

Several functional modules of proteins are involved in macroautophagic machinery:

(1) The autophagy-activating kinase complex ULK1/Atg1 forms a tetrameric complex with ATG13, ATG101, and FIP200, which is essential for autophagy induction (85); (2) The class III phosphatidylinositol 3-phosphate kinase (PI3K) complex 1, consisting of the VPS34 kinase subunit, VPS15(also known as PI3K regulatory subunit 4), Beclin 1/Atg6 and Atg14, that is required in early stages of autophagy (86); (3) Two ubiquitin-like conjugation systems Atg12-Atg5/Atg16L and microtubule-associated protein 1 light chain 3 (LC3)-Atg3, which are responsible for the formation and expansion of autophagosomes (87).

ULK1 is essential for recruiting class III PI3K complex 1 to the phagophore initiation site, thereby initiating vesicle nucleation. This PI3K complex produces phosphatidylinositol 3-phosphate (PI(3)P), which induces positive membrane curvature. Following this, the Atg12-Atg5/Atg16L complex is recruited to the phagophore, promoting LC3 lipidation. Lipidated LC3 binds to phosphatidylethanolamine (forming LC3-II), leading to autophagosome expansion and completion (88–92).

Besides non-selective manner, macroautophagy could also eliminate specific structures such as macroautophagy- mediated degradation of lipid droplets (lipophagy) or mitochondria (mitophagy) (10). While autophagy’s primary function is to protect cells from starvation and related stresses by breaking down macromolecules into basic building blocks for energy production, it also serves several other critical roles. For instance, in neurodegenerative diseases like Parkinson’s and Huntington’s diseases, autophagy helps by degrading α-synuclein and targeting mutant huntingtin proteins to clear aggregate-prone proteins. Autophagy also plays a protective role against certain infections caused by pathogens such as *Salmonella typhi* and *Mycobacterium tuberculosis*. In cancer, autophagy exhibits complex behavior: it may prevent the initiation of carcinogenesis but can also aid the survival of cells within nutrient-deprived solid tumors (93).

Besides the above functions, lysosomes also control regulate cell metabolism and are involved in various cellular processes such as secretion, plasma membrane repair, apoptosis and signal transduction

## 3 Pancreatic hormone & diabetes

### 3.1 Insulin & glucagon

Pancreatic islets are composed of several hormone-releasing cell types including α cells, β cells, δ cells, ϵ cells, and PP cells. It is known that different types of islet cells interact with each other via a paracrine mechanism. This paracrine signaling network is mediated by neighboring, cell contact via gap junctions, and local blood flow (94).

**Figure 3:**
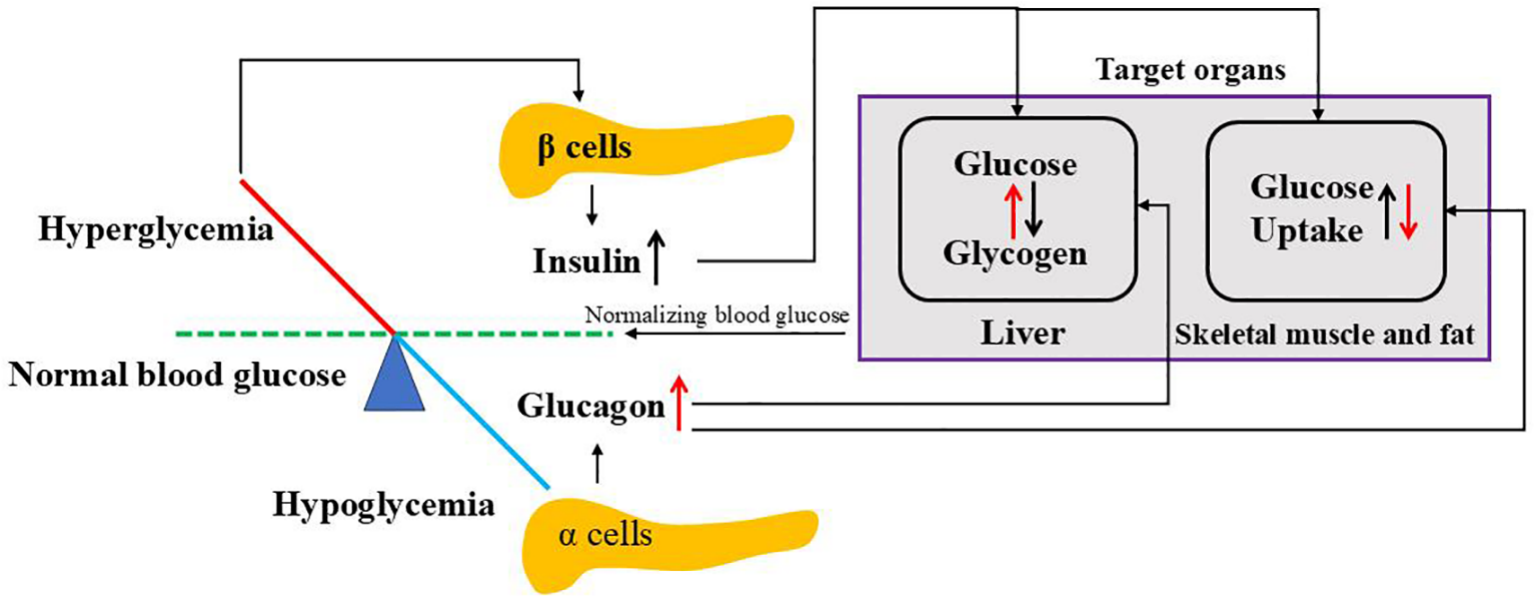
Regulation of blood glucose by insulin and glucagon. When blood glucose goes above the normal level, hyperglycemia stimulates pancreatic β cells to release insulin where it targets liver to convert glucose to glycogen and peripheral cells (skeletal muscle and fat) to uptake glucose. While blood glucose goes below the normal level, hypoglycemia triggers pancreatic a cells to release glucagon where it affects both liver and peripheral cells opposite to insulin. Both insulin and glucagon normalize blood glucose to normal ranges.

Insulin and glucagon, released by β and α cells of the pancreas respectively, work together to regulate blood sugar levels, ensuring a constant energy supply for the body. Glucagon, a catabolic hormone, promotes glycogenolysis and gluconeogenesis to increase the circulating glucose concentration upon blood glucose falls. Conversely, insulin, an anabolic hormone, reduces the plasma glucose concentration by promoting glucose uptake by cells, increasing glycogenesis, suppressing postprandial glucagon secretion and promoting protein and fat synthesis upon blood glucose is high (95) as shown in **Figure 3**.

#### 3.1.1 Glucagon release,secretion and biogenesis

Glucagon is a 29-amino acid peptide hormone, which is derived from the precursor proglucagon catalyzed by prohormone convertase 2 (PC2) in pancreas (96). Glucagon release is regulated through endocrine and paracrine pathways (97). Key regulators include free fatty acids, amino acids, neurotransmitters, and hormones such as insulin, somatostatin, and amylin, which can either stimulate or inhibit glucagon secretion. However, circulating glucose level is the most potent regulator of glucagon secretion. Low glucose level stimulates the pancreatic α cell to release glucagon while high glucose level inhibits glucagon secretion (97).

Glucose is taken up by the α cells through the glucose transporter 1 (GLUT1) and eventually converted by the mitochondria to ATP. Under conditions of high glucose concentrations, intracellular levels of ATP increase which closes ATP-sensitive potassium channel (K_ATP_) channels, thus leads to a strong depolarized membrane potential that inactivates voltage‐gated Na^+^ channels, resulting in reduced AP amplitude, less Ca^2+^ influx through P/Q‐type Ca^2+^ channels and thus suppressed glucagon secretion. When glucose concentration decreases, low intracellular ATP levels in α cell opens K_ATP_ channels with a low activity, which causes membrane depolarization to allow action potential firing. Then,the large amplitude action potentials activates P/Q‐type Ca^2+^ channels, allowing Ca^2+^ influx, which increases intracellular Ca^2+^ levels and triggers exocytosis of glucagon granules from α cells (98, 99). However, this process differs from that in β cells, as most KATP channels remain closed at low glucose concentrations in α cells (100).

#### 3.1.2 Insulin release, secretion and biogenesis

Insulin is a small protein composed of two polypeptide chains containing 51 amino acids. When plasma glucose level is high,extracellular glucose enters β cells via the glucose transporter glucose transporter 2(GLUT2)and is metabolized to produce ATP. The elevated ATP/ADP ratio results in the closure of K_ATP_ channels, influx of calcium through voltage gated Ca^2+^channels,exocytosis of insulin secretory vesicles and eventually the release of insulin (101).

Insulin is synthesized as a precursor at the ribosomes of the rough endoplasmic reticulum (RER) and is cleaved to proinsulin during its translocation into the lumen of the RER. Proinsulin molecules are then transported from the RER through the Golgi to the trans-Golgi network (TGN),where it is packaged into nascent insulin secretory granules (ISGs). Within these granules, proinsulin undergoes proteolytic cleavage by endopeptidases to produce insulin and C-peptide. Insulin is then condensed/crystallized with zinc and calcium, resulting in the formation of the mature dense-core granules. Finally, exocytosis of SGs upon stimulation results in insulin release (101).

High glucose stimulation can increase proinsulin synthesis by 50-fold at the translation level (102) and proinsulin is generally processed into insulin within 2 hours (101).

### 3.2 β cell function and diabetes

Plasma glucose concentration is maintained by the glucose entering the circulation and glucose removal from the circulation. The major sources of glucose are: intestinal absorption during the fed state, glycogenolysis (breakdown of glycogen) and gluconeogenesis (formation of glucose primarily from lactate and amino acids during the fasting state) (103).

Diabetes is the most common chronic disease worldwide. China, however, have the highest incidence of diabetes in the world and the prevalence of diabetes in the Chinese population has increased dramatically from 0.67% in 1980 to 11.6% in 2010, which is keeping on increasing (104). There are mainly two types of diabetes mellitus:

T1DM (type 1 diabetes mellitus) accounts for about 5–10% of patients with diabetes while T2DM accounts for about 90–95% of patients with diabetes (105).

T1DM typically begins in childhood and is an autoimmune disease in which the body’s immune system produces autoantibodies that attack the pancreatic β cells, leading to insulin production failure. The ideal therapy is the replacement of β cells by islet transplantation (106).

T2DM is usually due to inadequate insulin production and peripheral (happens in fat, liver, and muscle cells) insulin resistance with compensatory β cell expansion and hyperinsulinemia. Gradually decline in β cell function, reduction of glucose-stimulated insulin secretion (GSIS), decreased β-cell mass and increased β cell apoptosis have been found in human islets from patients diagnosed with T2DM (107). Patients with T2DM are at a significantly increased risk for complications, including heart disease, stroke, and high blood pressure (108). Unfortunately, T2DM is difficult to diagnose in its early stages, and there is currently no cure for the condition.

Treatment of T2DM focuses on improving insulin homeostasis and reducing peripheral insulin resistance. T2DM can be ameliorated through interventions such as enhanced diet and exercise, newer medications, or weight loss. Preserving insulin homeostasis and reducing peripheral insulin resistance remain the ideal goals for managing T2DM (105).

### 3.3 Glucagon function and diabetes

While insulin is the primary focus in diabetes treatment, the significance of glucagon should not be overlooked.

Hyperglycemia in T1DM is primarily attributed to the selective loss of β-cell mass, leading to reduced insulin secretion. However, emerging evidence suggests that glucagon plays an important role in the pathophysiology of T1DM. People with T1DM exhibit an impaired glucagon response to hypoglycemia, which increases the risk of severe hypoglycemia (109). In addition, excessive glucagon secretion may contribute to hyperglycemia, as elevated postprandial glucagon concentrations have been observed in T1DM patients (110). The underlying mechanism might be that in T1DM, the increase in plasma glucose after a meal does not trigger an increase in β cell insulin secretion, while in normal physiology, insulin secretion suppresses glucagon secretion. The loss of endogenous counterbalance from insulin may contribute to increased postprandial glucagon secretion from α cells (110). Given its role, glucagon is now being considered a potential therapeutic agent for treating hypoglycemia in individuals with T1DM (111).

In patients with T2DM, fasting glucagon is elevated, as well as glucose-induced glucagon suppression is impaired and postprandial insulin-glucagon interactions are disrupted,exhibiting an impaired regulation of glucagon secretion (112).

Considerable evidence suggests that hyperglucagonemia contributes to the hyperglycemic state in patients with T2DM (113, 114). Consequently, targeting glucagon has emerged as a promising strategy for improving glucose control in these patients. Dipeptidyl peptidase-4 inhibitors (DPP-4is) and glucagon-like peptide- 1 (GLP-1) receptor agonists (GLP-1Ras),which both suppress glucagon secretion, have attracted increasing attention to treat T2DM (112). Additionally, sodium-glucose cotransporter 2 (SGLT-2) inhibitors, a class of glucose-lowering drugs, reduce renal glucose reabsorption, promoting glucose excretion through urine and thereby lowering plasma glucose levels. These inhibitors may also have a direct effect on glucagon levels in patients with T2DM. Notably, the SGLT-2 inhibitors dapagliflozin and empagliflozin have been reported to increase both fasting and postprandial glucagon levels (115, 116).

## 4 Autophagy-lysosome pathway in insulin and glucagon homeostasis

### 4.1 Autophagy-lysosome pathway in insulin homeostasis

#### 4.1.1 Insulin production

Through a combination of metabolic labeling, immunoblotting, and immunohistochemistry techniques, researchers have identified the involvement of the lysosomal pathway in the early post-translational processing of proinsulin. During this stage, key processes such as ER translocation, structural maturation, and transport are regulated (117).

Recently, transcription factor EB (TFEB), a key regulator of lysosomal biogenesis, and its homolog TFE3 identified to regulate β function and insulin gene expression in response to variations in nutrient availability. Nutrient deprivation in β cells promotes TFEB/TFE3 activation, which in turn represses insulin gene expression while β cells lacking both TFEB and TFE3 are unable to repress insulin gene expression induced by amino acid deprivation (118).

#### 4.1.2 Insulin degradation

**Figure 4:**
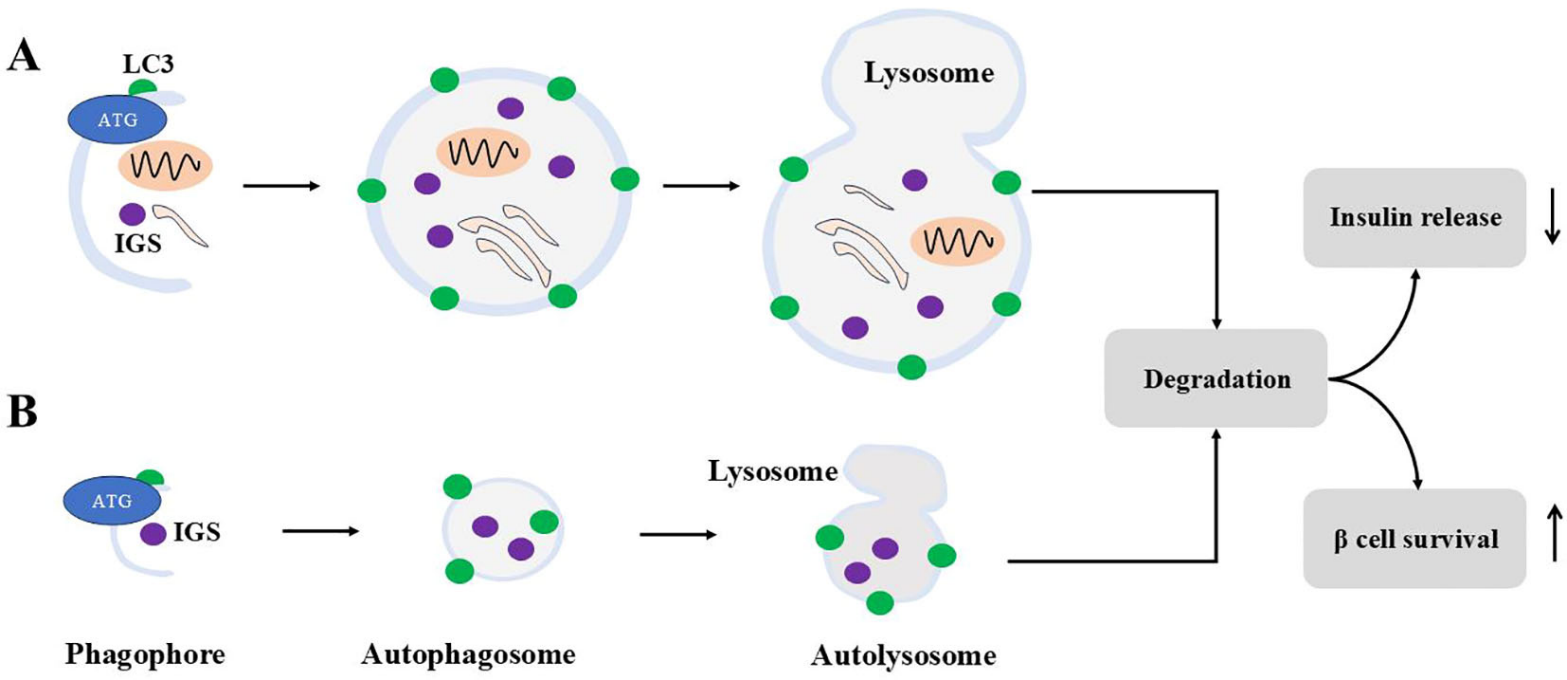
Macroautophagy and microautophagy for ISGs. Macroautophagy is shown in **(A)**, and **(B)** demonstrates microautophagy. For macroautophagy, autophagosomes engulf cytosolic contents including ISGs. For microautophagy, autophagosomes engulf ISGs only. Autophagosomes from both modes fuse with lysosomes to form autolysosome to degrade contents inside. Autophagy degradation reduces insulin release and sustains β cells survival under stress.

Insulin contents are regulated by crinophagy, a process where insulin granules fuse directly with vacuolar lysosomes to form crinophagic bodies, leading to granule degradation (119). This process is glucose-dependent: at low glucose levels, crinophagy increases to reduce intracellular insulin levels, whereas at high glucose levels, insulin degradation is inhibited (120) as **Figure 4** demonstrates. Insulin can also reach lysosomes through two additional pathways. In macroautophagy, autophagosomes engulf cytosolic components, including secretory granules, for lysosomal degradation. In microautophagy, lysosomes directly engulf individual granules. Interestingly, in β cells under starvation conditions, lysosomes degrade newly formed insulin granules. This degradation activates mTORC1, which suppresses autophagy and decreases insulin secretion during fasting (121). Consequently, insulin secretion decreases during fasting. Notably, stimulating autophagy has been shown to enhance insulin secretion in starved β cells. However, since starvation represents an extreme physiological condition, the broader significance of these findings for β cell function remains not clear.

The degradation of insulin precursor, proinsulin, involves macroautophagy. Confocal and electron microscopy have identified proinsulin within lysosomes and autophagosomes. Short-term treatment with bafilomycin A1, an inhibitor of vacuolar-type vacuolar type H(+)-ATPase, and knockdown of *Atg5* or *Atg7* significantly increased the steady-state levels of proinsulin and the hormone precursor chromogranin A (122). Furthermore, Rab7-interacting lysosomal protein (RILP), a key regulator of endosomal trafficking through its interaction with multiple Rab proteins, modulates insulin secretion by facilitating the lysosomal degradation of proinsulin. Overexpression of RILP induces the clustering of insulin granules and promotes proinsulin degradation, whereas depletion of RILP sustains proinsulin levels and enhances insulin secretion. Notably, RILP-induced proinsulin degradation is inhibited by lysosomal inhibitors and depends on Rab7 activity (123). Recently, vesicle-associated membrane protein 4 (VAMP4), a v-SNARE protein, has been identified as a regulator of insulin levels by facilitating the fusion of proinsulin granules with lysosomes under basal conditions (124).

Thus, it appears that insulin granule degradation is primarily mediated by microautophagy and crinophagy, while proinsulin degradation occurs via macroautophagy.

Recently, inceptor (insulin inhibitory receptor, encoded by the gene IIR/ELAPOR1) has been identified as a key player in insulin degradation. It localizes to clathrin-coated vesicles near the plasma membrane, the trans-Golgi network, and secretory granules, where it functions as a sorting receptor, directing proinsulin and insulin toward lysosomal degradation (125).

#### 4.1.3 Insulin release

Current evidence shows that the islet lysosome is also involved in the process of insulin secretion.

Glucose transporters (GLUTs) localized to the plasma membrane are rapidly degraded by lysosomes, a process induced by chronic high glucose or arginine stimulation. This degradation is driven by enhanced endocytosis caused by elevated extracellular glucose concentrations in β cells (13).

Several lysosomal enzymes and proteins are involved in the insulin secretion. Salehi A et al. reported that glucose-induced insulin release is dependent on islet acid glucan-1,4-a-glucosidase activity and normal function of the lysosomal/vacuolar system, while dysfunction of the islet lysosomal system impair glucose-induced insulin release in the diabetic GK rat (16).

Overexpression of the Iduronate-2-sulfatase (IDS), a lysosomal enzyme responsible for degradation of proteoglycans, potentiates the glucose-stimulated insulin secretory response by increasing the exocytosis through phosphorylation of PKCα and MARCKS (15).

Lysosomal lipid degradation, using lysosomal acid lipase(LAL) and potentially lipophagy, contributes to neutral lipid turnover in β cells and serves as a constitutive negative regulator of GSIS. Inhibition of LAL and autophagy *in vitro* and *in vivo* increase glucose-stimulated insulin secretion (14).

Gao et al. demonstrated that Sidt2, a lysosomal integral membrane protein, plays a vital role in regulating insulin secretion via the SNARE proteins synaptotagmin 1 (Synap1) and synaptotagmin 3 (Synap3). Depletion of Sidt2 in mice leads to weight loss, decreased survival with aging, elevated fasting glucose levels, impaired glucose tolerance, and reduced insulin levels. Additionally, INS-1 cells treated with Sidt2 siRNA exhibited decreased insulin production (12).

#### 4.1.4 Insulin sensitivity

Insulin sensitivity refers to the ability of cells—such as myocytes, hepatocytes, and white adipocytes—to respond effectively to insulin and mediate its actions. These actions include enhancing the absorption and storage of glucose and fatty acids.

A key player in insulin sensitivity is the insulin receptor (IR). Impaired activity of the receptor leads to insulin resistance, which is a key factor in the pathology of metabolic disorders such as T2DM. It is widely accepted that insulin resistance originates at the post-receptor level. Once activated, insulin receptors are internalized and transported to early endosomes, where they undergo dephosphorylation and sorting. Then, they can be transported to lysosomes for degradation or recycled back to the plasma membrane. Therefore, lysosomes play a role in insulin sensitivity that cannot be ignored (126).

Podocytes constitute the outer layer of the glomerular filtration barrier (GFB). The sensitivity of podocytes to insulin is key for the glomerulus to function normally. Impaired insulin signaling in podocytes can result in pathological features resembling diabetic nephropathy (DN). Research shows that saturated fatty acids can induce insulin resistance in podocytes (127, 128). Bafilomycin A1, an inhibitor of lysosomal acidification and protein degradation, was found to inhibit insulin-dependent IR turnover and reduce insulin-dependent glucose uptake in cultured rat podocytes. These findings suggest that lysosomes may be involved in regulating IR signaling and glucose uptake in podocytes. Disruptions in insulin signaling to lysosomes can impair lysosomal activity, contributing to the development of insulin resistance in podocyte (129).

Studies using Becn1^F121A^ knock-in mice, which exhibit constitutively active autophagy, show that autophagy selectively sequesters and degrades insulin granule vesicles in β cells, leading to decreased insulin secretion and storage. However, insulin sensitivity in insulin-responsive tissues (skeletal muscle, liver, and white adipose tissue) improves due to reduced ER stress under a high-fat diet (79).

Recently, it has been reported that elevated hepatic SLC7A14 induces insulin resistance, working as a transporter for importing GABA to lysosomes. Thus, the increased lysosomal GABA mediates SLC7A14-induced insulin resistance.mTORC2 is involved in lysosomal GABA accumulation-induced insulin resistance (130).

Interestingly, the loss of lysosomal acid lipase (LAL), an enzyme that hydrolyzes cholesteryl esters and triacylglycerols (TG), has been shown to decrease VLDL secretion while increasing insulin sensitivity. Mice lacking LAL exhibit enhanced glucose clearance during insulin and glucose tolerance tests and demonstrate increased glucose uptake into skeletal muscle compared to wild-type mice (131).

Moreover, TFEB, a key regulator of lysosomal biogenesis, and its induced expression of GDF15, may serve as a lysosomal response mechanism. Together, they potentially protect against obesity and insulin resistance (132).

### 4.2 Autophagy affects glucagon homeostasis

The association between autophagy and glucagon was reported approximately 60 years ago. In 1962, Ashford and Porter (133) found that glucagon administration increased the autophagy in liver.

Glucagon binds to glucagon receptor (a G protein-coupled receptor) on the hepatocyte stimulating cAMP productions by adenyl cyclase. Increase in the intracellular level of cAMP activates protein kinase A (PKA) and inhibits salt-inducible kinases (SIK). Then PKA phosphorylates Ser133 of cyclic AMP-responsive element-binding protein (CREB) and SIK dephosphorylates Ser171 of CREB-regulated transcription co-activator (CRTC). Phosphorylated CREB together with CRTC upregulates CREB target genes such as the gluconeogenesis-related genes PGC1α, nuclear receptor subfamily 4 group A member 1 (NR4A1) and TFEB which regulates gene expressions of autophagy proteins (8).

Yang et al. found that O-linked β-N-acetylglucosamine (O-GlcNAc) transferase (OGT), proposed to function as a nutrient sensor, is essential for glucagon-induced autophagy in the liver during starvation. In this process, glucagon triggers calcium signaling, which leads to CAMKII-mediated phosphorylation of OGT. This, in turn, facilitates Ulk O-GlcNAcylation and activation, ultimately initiating autophagy (134).

However, although numerous studies support the role of glucagon in inducing autophagy (133, 135, 136), fewer have explored how autophagy regulates glucagon.

Autophagy plays a role in maintaining α cell and normal islet architecture but appears to be dispensable for metabolic homeostasis as cellular proliferation was suppressed in *Atg7*(an essential gene for autophagosome formation)-deficient α cell but plasma glucagon levels, which were measured after an 8-hour fast or after insulin injection during the ITT(Insulin tolerance tests), were not different between a *Atg7* KO and control at the age of 10 weeks (21). Kim et al. reported that autophagy-deficient mice exhibit impaired incretin-induced suppression of glucagon release from α-cells following glucose loading, a process potentially mediated by cAMP (20). Furthermore, amino acid starvation reduces glucagon secretion via mTORC1 inhibition and crinophagy-mediated degradation of glucagon, a process distinct from macroautophagy (19). Conversely, amino acids promote glucagon secretion by activating mTORC1 in α-cells. Mice with α-cell-specific raptor loss exhibit normal cell mass but defective glucagon maturation and secretion (137). Glucagon also induces proteolysis. T2DM patients have elevated glucagon level which result in elevated amino acid level to induce α cell hyperplasia by an mTORC1 dependent mechanism (138–140). In addition to autophagy, endosomes play a significant role in glucagon degradation in intact rat liver, a process linked to ATP-dependent endosomal acidification (141, 142).

These findings collectively suggest that the lysosome regulates glucagon homeostasis, though further studies are required to form a comprehensive understanding of the mechanisms involved.

## 5 Lysososome-autopahgy and diabetes

### 5.1 Autophagy is required to maintain normal function of β cell

A relatively low level of autophagy is essential for maintaining the physiological structure and function of pancreatic β cells. Ebato et al. found that autophagosomes were barely detectable in the β cells of standard-fed mice due to their small size. In contrast, Atg7-deficient mice exhibited suppressed LC3-I to LC3-II transition, along with the accumulation of p62 and polyubiquitin. Additionally, the islets in these mice contained multiple cyst-like structures measuring 15–20 µm in diameter (17).

*In vivo* studies demonstrated that LC3-I and LC3-II levels significantly increased when INS-1 cells were treated with 100 nM insulin for 24 hours, compared to controls, suggesting that high insulin levels upregulate autophagy. Treatment with 3-MA, an autophagy inhibitor, markedly increased apoptosis compared to cells treated only with insulin, underscoring the critical role of autophagy in maintaining β cell viability (143).

In the context of β cell adaptation to metabolic stress, such as high-fat diet (HFD) feeding, mitophagy is induced. This process is mediated by lysosomal Ca^2+^ release, leading to increased cytosolic calcium concentration and subsequent activation of TFEB, facilitating cellular adaptation to metabolic stress (144, 145).

### 5.2 Autophagy in β cells in response to cellular stress linked to T2DM

Mounting studies have demonstrated that macroautophagy maintains β cell function in cellular stress linked to T2DM, found in lipo- and glucotoxicity models (146–148), Endoplasmic Reticulum (ER) stress induced diabetes (149, 150) and human islet amyloid polypeptide induced diabetes (151, 152).

Though deletion of *Atg7* was not sufficient to induce diabetes,it could induce glucose intolerance,impaired insulin secretion especially when fed a high fat diet in mouse (17, 153). Interestingly, in leptin-deficient ob/ob mice, a genetic model of obesity, the absence of Atg7 resulted in severe diabetes (150). However,these results are not consistent with Riahi Y’s results that *Atg5/7* knockdown increased glucose- and non-fuel-stimulated insulin secretion (122). Additionally, long-term HF diet induced T2DM in zebra fish and Palmitic acid induced type 2 HepG2 cells showed decreased autophagy as evidenced by reduced transcription of autophagy-related genes (*Atg*3, *Atg*4B, *Atg*5, *Atg*7, *Atg*12, and FOXO3) and increased expression of the autophagy inhibitor mTOR. In the liver of HFD-fed zebrafish, autophagy flux was inhibited, while the conjugation of preproinsulin with the cargo-recognition protein p62 increased. However, the formation of autophagosomes, lysosomes, and autolysosomes with p62-cargo decreased significantly (154).

High glucose can induce autophagy MDC (monodansylcadaverine)- labelled autophagosomes and LC3-II expression were increased upon glucose treatment compared with those of the control group. Inhibiting autophagy with 3-MA reduced the viability of islet β cells compared to cells treated only with high glucose. These findings suggest that autophagy induction under high glucose conditions protects islet β cells from death. Elevated glucose increases insulin demand, stimulating proinsulin synthesis in β cells. Autophagy facilitates protein delivery, and its inhibition may result in the accumulation of misfolded proteins, inducing ER stress and leading to cell death (155).

However, the duration of glucose and/or palmitate exposure influences macroautophagy. Short-term exposure enhances macroautophagy, while long-term exposure diminishes it (148, 156–162). Altered levels of macroautophagy have been reported in isolated islets and cellular models where the diabetic environment was mimicked to a certain degree by adding high amounts of glucose and/or free fatty acids such as palmitate. For example, in the β-cell line INS-1E, LC3-II levels increased with palmitate treatment up to 8 hours, followed by a decrease. In isolated human islets, LC3-II signals increased up to 48 hours (161). Similarly, INS-1E cells showed a time-dependent increase in autophagic flux upon palmitate treatment (up to 8-16 h), followed by a decrease (at 24 h) as compared to control- treated cells (157).

Altered autophagic activity has been detected in β cells of T2DM patients. These patients exhibit an increased fraction of β cells overloaded with vacuoles and a higher volume density of autophagic structures (163). Elevated levels of the macroautophagy substrate p62 (164–166) and reduced LC3-II levels (164) in β cells suggest decreased macroautophagy in T2DM.

Notable, although the role of autophagy in T1DM is less well studied than in T2DM, defective islet autophagy indeed contributes to β-cell dysfunction in the pathogenesis of T1DM (194). *Clec16a*, a T1DM susceptibility gene, has been demonstrated to play a critical role in regulating mitophagy, glucose-stimulated insulin secretion (167) and protection of β-cells against cytokine-induced apoptosis in type 1 diabetic mouse (168, 169). Expression of this gene was controlled by β cell transcription factor, PDX1, through its regulation of the E3 ubiquitin ligase neuregulin receptor degradation protein (Nrdp)1 (169).

### 5.3 Key lysosomal proteins (complex) in regulation of β cell structure & function and diabetes

#### 5.3.1 mTOR

mTORC1 signaling in β cells plays a critical role in maintaining systemic glucose homeostasis by regulating several key processes, including β cell mass, proliferation, apoptosis, insulin secretion, and the degradation of insulin secretory granules (170). Mice deficient in mTORC or Raptor specifically in β cells or pancreatic progenitor cells showed decreased β cell mass, defective islet development, hypoinsulinemia and glucose intolerance (170–172). In Akita mice, stimulation of β cell macroautophagy by rapamycin-mediated inhibition of mTOR prevented β cell apoptosis, increased pancreatic insulin content and ameliorated diabetes (149).

The effect of mTORC1 on β cell survival is biphasic. Short-stimulus or transient activation of mTORC1, the conditions such as fasting and re-feeding or short-term nutrient or growth factor stimulation under physiological environment, increase protein synthesis and insulin secretion. However, sustained activation, as seen in over-nutrition or hyperinsulinemia, correlates with cell death and impaired glucose-stimulated insulin secretion. Glucose promotes rodent and human β cell proliferation via mTORC1 under physiological conditions (173, 174). Yet, chronic mTORC1 hyperactivation due to prolonged high glucose exposure impairs β cell survival in mouse islets and clonal β cells (175, 176). In Min6 clonal β cells, short-term palmitate exposure increases mRNA translation by promoting polyribosome occupancy, but prolonged exposure depletes polyribosome-associated RNA and triggers ER stress. These effects are mediated by mTORC1, as its inhibition mitigates both the acute translation increase and chronic ER stress (177).

Studies on β cell specific TSC2 deletion (β-TSC2-KO) have shown conflicting results. Sustained mTORC1 activation increases islet mass due to enhanced β-cell number and size in young mice (up to 30 weeks), but older mice experience β cell loss due to apoptosis, leading to diabetes (178, 179). Interestingly, β-TSC2-KO mice exhibit chronic hyperinsulinemia for up to 10 months, which may induce ER stress from increased proinsulin biosynthesis, resulting in β cell death (180). However, some studies did not observe this biphasic effect (181).

For S6K1, β cell specific S6K1overexpression shows improved insulin secretion, but cell cycle progression is impaired, and apoptosis increased, with consequent reduction of β cells per islet. However, total β cell mass is not affected due to β cell hypertrophy. Furthermore, in isolated islets from β cell-specific S6K1-overexpressing mice, glucose-induced ATP induction is blunted, together with impaired glucose-stimulated insulin secretion (182). β Cells from S6K1–/– embryos exhibit reduced cell size and insulin content, however, re-expression of S6K1 in β cells of S6K1–/– mice restored embryonic β cell size, insulin levels, glucose tolerance, and RPS6 phosphorylation (183). Activation of MTORC1 by TSC1 is also reported to induce β cell hypertrophy and hyperinsulinemia (184).

For diabetes, or conditions of cellular nutrient overload relevant to T2DM, mTORC1 is upregulated in β cells while its inhibition prevents β cell death and enhances insulin secretion (148, 175–179, 185–187). Rapamycin-mediated stimulation of macroautophagy increased insulin secretion and decreased apoptosis in human islets isolated from T2D patients (188). Disruption of mTORC1 activity by silencing Raptor was shown to enhance glucose-stimulated insulin secretion in a rodent β cell line. This augmentation is achieved by transcriptional upregulation of Ins1 and Ins2 mRNA as well as higher insulin production increasing PDX1 and MafA expression, two transcription factors that control insulin production to prompt insulin production (189). In rodent INS-1E cells, the cytokine IL-6 is crucial for promoting protective autophagy, which helps cells resist apoptosis, by primarily working through the inhibition of mTORC1 signaling pathway (190) while it also reduce couples autophagy to antioxidant response and thereby reduces β-cells and human islets oxidative stress (191).

The importance of mTORC1- mediated suppression of macroautophagy in the β cell is further reflected by the fact that the recently discovered mTORC1-independent macroautophagy inducer MSL-7 had virtually no effect in the β cell of high fat diet-fed mice, while being a potent activator of macroautophagy in liver and adipose tissue (192).

#### 5.3.2 TPC channels

TPCs are ion channels that release calcium from intracellular compartments when activated by specific signaling molecules. NAADP-evoked Ca^2+^ release from acidic Ca^2+^ storage organelle activates inward membrane currents, depolarize the β cell and thereby contributes to glucose-evoked insulin secretion. The whole pancreas derived from TPC1 knock-out mice secrete less insulin in response to elevated glucose (193). However, Ca^2+^ release from acidic stores through TPC1 might be sufficient to support normal Ca^2+^ dynamics in response to stimulation by nutrients or incretins while TPC2 is not absolutely required for normal glucose- or incretin-stimulated insulin secretion from the β cell as β cell specific knockout of TPC2 did not affect glucose-evoked Ca^2+^ signaling, insulin secretion or glucose tolerance (194). One possibility to explain these different results is that TPC knockout is compensated for *in vivo*.

Since TPCs regulate autophagy, alterations in TPC activity can affect glucose metabolism by influencing autophagic processes (195, 196).

Adrenaline is a potent stimulator of glucagon secretion. Inhibition of TPC2 abolishes the stimulatory effect of adrenaline on glucagon secretion and reduces the elevation of intracellular calcium levels in α cells. This highlights the regulatory role of TPC2 in glucagon secretion (197).

The exact mechanisms by which TPC1 and TPC2 influence glucose regulation are still being investigated, and further research is needed to fully understand their role in different cell types and physiological conditions.

#### 5.3.3 P2X4 channels

P2X4 has been found expressed in mouse islet and Β-TC6 cells. P2X4 receptor potentiator ivermectin augmented GSIS and slightly potentiated the effect of ATP stimulated insulin secretion by islets in the presence of high glucose. P2X4 is involved in the inhibitory effect of ATP on cell proliferation (57). P2X4 can also moderate high glucose and palmitate induced inflammatory responses in endothelial cells and endothelial dysfunction is an early determinant of the progression of vascular disease, which is common in the pathogenesis of diabetes and cardiovascular disease (198).

#### 5.3.4 TRPML channels

RNA sequence analysis revealed that TRPML1 and TRPML3 are expressed in human β cells (199). Furthermore, TRPML1 is expressed at higher levels in human β cells compared to α cells (200). Significant reductions in the gene expression of TRPML2 and TRPML3 were observed in patients with obesity-related metabolic syndrome. However, direct evidence linking TRPML2 and TRPML3 to insulin homeostasis and diabetes remains lacking (59). Recently, it was reported that perfluorooctane sulfonate (PFOS) induces autophagy-dependent calcium overload in lysosomes, leading to the transfer of lysosomal calcium to mitochondria. This lysosome-to-mitochondria calcium transmission is mediated by the plasma membrane ATP synthase F1 subunit β (ATP5B), which regulates the interaction between TRPML1 and the voltage-dependent anion channel 1 (VDAC1). This mechanism promotes hepatic insulin resistance under PFOS exposure (201).

#### 5.3.5 TRPM2 channels

TRPM2 has been shown to function as a Ca^2+^-release channel in the lysosomes of β cells (63). Since intracellular calcium elevation is essential for insulin secretion, substantial evidence suggests that TRPM2 channels play a role in this process. Co-expression of TRPM2 with insulin has been observed, and activation of these channels increases in both cytosolic Ca^2+^ and insulin release (66). TRPM2 knockout mice exhibit impaired insulin secretion and elevated blood glucose levels in glucose tolerance tests. Insulin secretion from the islets of TRPM2 knockout mice, in response to glucose and incretin hormone treatment, is also reduced. Furthermore, in isolated β cells, a decreased intracellular calcium increase is observed in response to high concentrations of glucose and incretin hormones in TRPM2 knockout mice (202). In isolated pancreatic islet cells from TRPM2 knockout (KO) mice, compared to wild-type (WT) mice, H_2_O_2_-induced calcium influx and the enhancement of insulin secretion are both reduced (203). Lysosomal Ca^2+^ release through TRPM2 also contributes to H_2_O_2_-induced apoptosis in β cells (63, 204). Moreover, the incretin hormone, GLP-1, enhanced GSIS is also reported to act via TRPM2 (202, 205, 206). GLP-1 receptor stimulation increases TRPM2 channel activity through the cAMP-EPAC pathway (206). Zhang et al. found that TRPM2 deletion protects mice from developing insulin resistance and obesity induced by a high-fat diet. TRPM2 knockout mice display enhanced insulin sensitivity, partly due to increased glucose metabolism in peripheral organs. Their resistance to high-fat diet-induced obesity is associated with enhanced energy expenditure, and the obesity-related inflammation in adipose tissue and liver is attenuated (207). Inhibition of TRPM2 channels may offer a therapeutic strategy for treating endothelial dysfunction associated with oxidative stress, as TRPM2-activated Ca^2+^ signaling is necessary for inducing endothelial insulin resistance in obesity (208).

### 5.4 Autophagy as therapeutic target in diabetes

Autophagy plays a critical role in regulating obesity, diabetes, and their complications, rendering it a potential therapeutic target.

Cheng et al. discovered that a fasting-mimicking diet could promote β cell regeneration in a T2DM model (209). This effect can be replicated by suppressing autophagy inhibitors such as mTORC1 or PKA, indicating that normal autophagy is essential for maintaining islet β cells and insulin production. Intermittent fasting has been shown to promote β cell neogenesis through autophagy induction (209–211). However, this process was abrogated when autophagy is suppressed, indicating autophagy may play an important role in intermittent fasting induced β cell neogenesis. In line with this notion, recent studies have proposed to improve lysosomal function in β cell by using bioengineered nanoparticles designed to lower lysosomal pH (212, 213).

Of note, several drugs that known to treat diabetes could increase β cell macroautophagy: (1) metformin, widely used in T2DM, improves macroautophagy in β cells via the AMPK pathway (214). (2) Rosiglitazone stimulates autophagy and protects β cells from fatty acid-induced cell death, also via the AMPK pathway (215). 3) GLP-1 receptor agonists like liraglutide and exendin-4 promote macroautophagy in INS1 cells, isolated human islets, and rodent models of diabetes (216–218). Notably, exendin-4 improves β-cell function in macroautophagy-deficient β cells and Atg7 β-cell KO mice by enhancing insulin secretion and reducing apoptosis (109). 4) DDPP-4 (Dipeptidyl peptidase-4) inhibitors, such as MK-626, increase macroautophagy in islets from high-fat diet-fed mice (219).

Interestingly, a macroautophagy-independent lysosomal degradation of newly formed insulin granules has been reported in different model systems of diabetes including human origin (β cell lines, human and mouse pancreatic islets as well as diabetic mice.). This pathway happens under stress condition referred to as stress-induced nascent granule degradation(SINGD) and contributes to loss of insulin as well as mTOR-dependent suppression of autophagy. Protein Kinase D (PKD), as a negative regulator of SINGD, is reported reduced in diabetic β Cells. Activation of PKD inhibits SINGD and postpones the T2DM.Their findings provide a new therapeutic strategy to treat T2DM (121, 220).

### 5.5 Lysosomal proteins and autophagy is required to maintain normal structure and function of α cell

The depletion of *Atg7* in α cells in mice suppresses α cell proliferation, despite an observed increase in the ratio of α cells. This suggests that autophagy in α cells plays a critical role in maintaining normal islet architecture (221).

Interestingly, the depletion of *Atg7* in β cells does not affect α cell morphology or mass. However, it does eliminate the incretin-induced suppression of glucagon release from α cells (222).

### 5.6 Autophagy in α cells as therapeutic target in α cell in diabetes

As mentioned before, hyperglucagonemia is a key contributor to hyperglycemia in diabetes patients (110, 113, 114). This condition arises from dysregulated glucagon secretion by pancreatic α cells (223). One potential mechanism underlying this dysregulation is impaired intracellular trafficking of glucagon (224). Recent studies have identified altered lysosomal trafficking of glucagon as a novel pathway contributing to excess glucagon in diabetes (225, 226). Specifically, a shift occurs from transport lysosomes (Lamp-2A+) to autolysosomes and subsequently to secretory lysosomes (Lamp-1+), accompanied by increased glucagon trafficking through secretory granules (225).

## 6 Conclusion

Autophagy is important for maintaining insulin and glucagon homeostasis and plays an important role in protecting α and β cells,targeting its proteins and transporters may be a promising therapeutic approach.

Impairment of autophagy induces a diabetic- like state and altered autophagy is also seen in human from diabetic subjects compared to controls.

It is likely that targeting of altered autophagy will have significant benefits in the treatment of diabetes or even in the prevention of the progression of obesity to T2DM. However, the development of therapies will require a much greater understanding of the complex role of autophagy in diabetes and obesity.
